# Neuroendocrine Carcinomas of the Uterine Cervix, Endometrium, and Ovary Show Higher Tendencies for Bone, Brain, and Liver Organotrophic Metastases

**DOI:** 10.3390/curroncol29100587

**Published:** 2022-10-06

**Authors:** Hyung Kyu Park

**Affiliations:** Department of Pathology, Chungnam National University School of Medicine, Daejeon 35015, Korea; hkpark@g.cnu.ac.kr

**Keywords:** neuroendocrine, cervical cancer, endometrial cancer, ovarian cancer, metastasis

## Abstract

Neuroendocrine carcinoma (NEC) of the female genital tract is a rare and aggressive subtype of cancer that is still poorly understood. Several recent studies reported that pulmonary and gastroenteropancreatic neuroendocrine neoplasms show significantly different patterns of metastasis compared to non-NECs of the same primary sites. The aim of this study was to evaluate the metastatic patterns of gynecologic NECs and to compare the metastatic patterns of NECs and non-NECs of the same primary sites. We retrieved and analyzed cervical, endometrial, and ovarian NEC cases from the Surveillance, Epidemiology, and End Results (SEER) database. To validate the results, we also retrieved and analyzed cervical NEC cases from an institutional database. Uterine cervical NEC was the most common NEC. The overall metastatic rate was significantly higher in the NEC group than in the non-NEC group for all three primary sites. All cervical, endometrial, and ovarian NECs showed a higher tendency for bone, brain, and liver organotrophic metastasis than non-NECs of the same primary sites. We demonstrated that gynecologic NECs show significantly different metastatic patterns compared to non-NECs of the same primary sites. These findings might help clinicians to better manage patients with gynecologic NECs.

## 1. Introduction

Neuroendocrine carcinoma (NEC) is a subgroup of epithelial neuroendocrine neoplasms (NENs) and is known to show aggressive clinical behavior [1]. NEC can occur in various organs. However, NEC predominantly occurs in a few selected organs, such as the lung and gastrointestinal (GI) tract, and it only rarely occurs in other anatomic sites, including the uterus and ovaries [2]. Consequently, most previous studies have focused on NECs originating from particular organs, such as the lung [1]. There are few previous studies on NECs in the female genital tract, which has led to difficulties in the management of such patients.

Some recent studies have reported that pulmonary and GI NENs, including NECs, show significantly different patterns of metastasis compared to non-neuroendocrine carcinomas (non-NECs) of the same primary sites [3,4]. If pulmonary and GI NECs show significantly different patterns of metastasis, NECs in the female genital tract might also show significantly different patterns of metastasis compared to non-NECs of the same primary sites. Considering that NECs are known to show aggressive behavior and frequent metastasis, understanding the exact metastatic pattern of NEC would be important for proper patient management.

Unfortunately, it is very difficult to collect a sufficient number of patients with gynecologic NECs for statistical analysis, due to its rarity. To solve this problem, we thought that analyzing the data provided by the Surveillance, Epidemiology, and End Results (SEER) program might be a valuable option. However, the SEER database only includes information on metastasis at the time of diagnosis, as well as several other limitations. Therefore, we aimed to compare the metastasis patterns of NECs and non-NECs of the uterine cervix, endometrium, and ovaries using the SEER database, and then validate the results using an institutional database to determine whether gynecologic NECs show significantly different metastasis patterns compared to non-NECs of the same primary sites.

## 2. Materials and Methods

### 2.1. SEER Database

The datasets were retrieved from the “Incidence—SEER Research Data, 17 Registries, Nov 2021 Sub (2000-2019)” database provided by the SEER Program using the Case Listing Session of SEER*Stat software version 8.4.0.1 [5,6]. The case selection criteria were as follows: (a) primary carcinomas of the uterine cervix, uterine corpus, and ovaries, (b) diagnosis obtained between 2010 and 2019, and (c) histologic diagnosis of NECs and representative non-NECs of each primary organ. For the representative non-NECs, we selected adenocarcinoma (ADC) and squamous cell carcinoma (SCC) in the uterine cervix, and endometrioid carcinoma (EC), mucinous carcinoma (MC), and serous carcinoma (SC) in the endometrium and ovary. To select cases in which a proper histologic diagnosis was obtained, we used the International Classification of Diseases for Oncology, 3rd Edition (ICD-O-3) histology codes. We did not include cases of histologically combined tumors. Then, we extracted the following data: patient IDs, year of diagnosis, ICD-O-3 histology codes, M stage, and data regarding specific metastasis sites, including bone, brain, liver, lung, distant lymph nodes (LNs), and other sites. Cases with an unknown M stage were excluded.

### 2.2. Institutional Database

We searched an institutional database and retrieved data from patients diagnosed between 1994 and 2014. Except for the year of diagnosis, we used the same case selection criteria that were used to search the SEER database. Patients with histologically combined carcinoma were also excluded. Then, we extracted the clinical data, including age at diagnosis, histologic diagnosis, presence of metastasis, and metastatic sites.

### 2.3. Statistical Analysis

Categorical variables such as the presence of metastasis were compared using Pearson’s chi-squared test or Fisher’s exact test, as appropriate. We performed statistical analyses using SPSS version 17.0 (SPSS Inc., Chicago, IL, USA).

## 3. Results

### 3.1. SEER Database

Among the uterine cervical carcinomas, we identified and retrieved data on 495 NECs and 26,464 non-NECs (19,947 SCCs and 6517 ADCs). Among the endometrial carcinomas, we identified and retrieved data on 173 NECs and 100,526 non-NECs (89,637 ECs, 702 MCs, and 10,187 SCs). Among the ovarian carcinomas, we identified and retrieved data on 242 NECs and 31,787 non-NECs (4958 ECs, 2700 MCs, and 24,129 SCs). Uterine cervical NEC was the most common NEC, followed by ovarian NEC and endometrial NEC.

The proportion of patients who presented with metastasis at the time of diagnosis was significantly higher in the NEC group than in the non-NEC group for all three primary sites. Specifically, 43.2% (214/495) of the patients with cervical NEC had metastasis at the time of diagnosis, which was significantly higher (all *p* < 0.001) than the corresponding percentages of patients with cervical SCC (12.9%, 2569/19,947) and cervical ADC (10.3%, 674/6517). Similarly, 44.2% (73/165) of patients with endometrial NEC had metastasis at the time of diagnosis, which was significantly higher (all *p* < 0.001) than the corresponding percentages of patients with endometrial SC (22.6%, 2291/10,152), endometrial MC (4.7%, 33/699), and endometrial EC (3.1%, 2,725/89,334). In addition, 40.1% (87/217) of patients with ovarian NEC had metastasis at the time of diagnosis, which was also significantly higher (*p* = 0.007, *p* < 0.001, and *p* < 0.001, respectively) than the corresponding percentages of patients with ovarian SC (31.5%, 7563/24,028), ovarian MC (10.2%, 274/2687), and ovarian EC (5.2%, 259/4946).

When we compared metastatic rates of NECs and non-NECs of the same T stage, all of the cervical, endometrial, and ovarian NECs also showed higher metastatic rates than those of non-NECs of the same T stage. In detail, cervical T1b NEC showed a higher metastatic rate (18.4%, 26/141) than those of T1b SCC (3.8%, 215/5639) and T1b ADC (3.4%, 101/2937). Ovarian T1 NEC showed a higher metastatic rate (20.6%, 13/63) than those of T1 SC (8.7%, 229/2637), T1 MC (1.2%, 24/1946), and T1 EC (1.1%, 36/3244). Similarly, endometrial T1 NEC showed a higher metastatic rate (26.4%, 14/53) than those of T1 SC (5.2%, 277/5322), T1 MC (0.3%, 2/572), and T1 EC (0.6%, 454/75460). Because of the small number of patients with ovarian or endometrial NEC, we were not able to compare metastatic rates of ovarian and endometrial T1b NECs with T1b non-NECs of the same primary sites.

**Figure 1:**
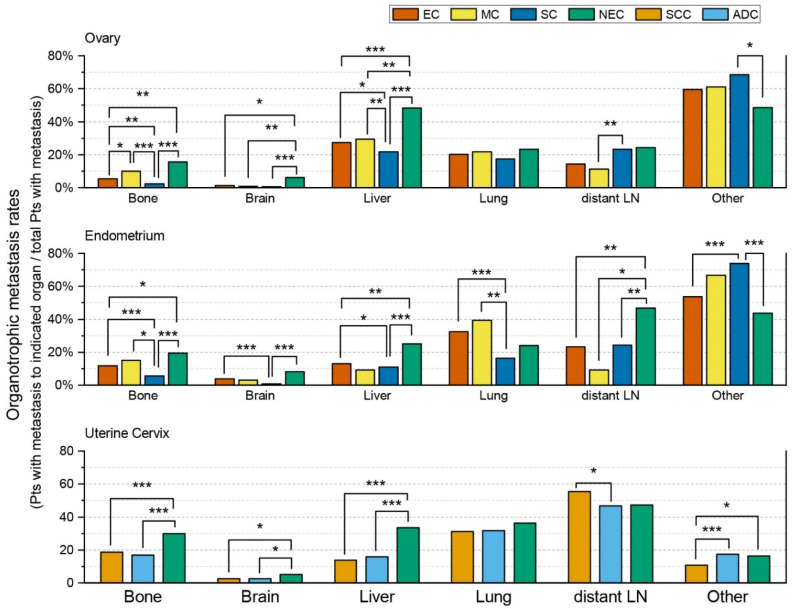
Comparison of organotrophic metastasis patterns between neuroendocrine carcinomas and representative non-neuroendocrine carcinomas of the uterine cervix, endometrium, and ovaries based on cases retrieved from the Surveillance, Epidemiology, and End Results (SEER) database. Only statistically significant comparisons are marked with asterisks (*, *p* ≤ 0.05; **, *p* ≤ 0.01; ***, *p* ≤ 0.001). (Abbreviations: EC, endometrioid carcinoma; MC, mucinous carcinoma; SC, serous carcinoma; NEC, neuroendocrine carcinoma; SCC, squamous cell carcinoma; ADC, adenocarcinoma; Pts, patients; LN, lymph node).

As we described above, there were significant differences in the overall metastatic rates (percentage of patients with metastasis/total number of patients) among different histologic types of carcinomas. We considered that these differences could cause statistical biases during the comparison of metastatic patterns between NECs and non-NECs. To avoid such biases, we calculated and compared the organotrophic metastasis rates (the percentages of patients with metastasis of the indicated organ/the total number of patients with metastasis) during comparisons of the metastatic patterns between NECs and non-NECs. The results are illustrated in Figure 1.

Cervical NEC showed significantly higher bone, brain, and liver organotrophic metastasis rates than cervical non-NECs, including SCC and ADC (Table 1 and Table S1). Additionally, cervical NEC showed a significantly higher organotrophic metastasis rate to other organs than cervical SCC. However, cervical NEC showed a similar organotrophic metastasis rate to other organs compared to cervical ADC. Lung and distant LN organotrophic metastasis rates also did not show statistically significant differences between cervical NEC and cervical non-NECs. In contrast, cervical SCC and ADC did not show significantly different bone, brain, liver, and lung organotrophic metastasis rates. However, cervical SCC showed a significantly higher distant LN organotrophic metastasis rate than ADC. ADC had a significantly higher organotrophic metastasis rate to other organs than SCC. 

Ovarian NEC showed significantly higher brain and liver organotrophic metastasis rates than ovarian non-NECs, including EC, MC, and SC (Table 2). Ovarian NEC showed a significantly higher bone organotrophic metastasis rate than ovarian EC and SC. The bone organotrophic metastasis rate was also higher for ovarian NEC than for ovarian MC. However, the difference was not statistically significant. In contrast, there were no statistically significant differences in lung and distant LN organotrophic metastasis rates between ovarian NEC and non-NECs. The organotrophic metastasis rates to other organs were also not significantly different among ovarian NEC, EC, and MC. However, the organotrophic metastasis rate to other organs was significantly lower for ovarian NEC than for ovarian SC. Interestingly, bone organotrophic metastasis rates were significantly different among ovarian non-NECs (Table S2). This rate was significantly higher in MC (10.0%, 26/260), followed by EC (5.3%, 13/247), and SC (2.2%, 157/7293). The organotrophic metastasis rates to the brain, lung, and other organs were similar among ovarian non-NECs. The liver organotrophic metastasis rate was significantly lower for SC than for EC or MC.

Because we were only able to retrieve 72 cases of endometrial NEC with metastasis and 33 cases of endometrial MC with metastasis, statistical analyses were limited, especially when comparing endometrial NEC and MC. Endometrial NEC showed higher bone, brain, and liver organotrophic metastasis rates than endometrial MC (Table 2 and Table S3). However, the differences were not statistically significant. Endometrial NEC showed significantly higher bone and liver organotrophic metastasis rates than endometrial EC and SC. Endometrial NEC also showed a higher brain organotrophic metastasis rate than endometrial EC and SC. However, this difference was only statistically significant between endometrial NEC and SC. Additionally, endometrial NEC showed a significantly higher distant LN organotrophic metastasis rate than endometrial non-NECs. Lung organotrophic metastasis rates were not significantly different between endometrial NEC and non-NECs. The organotrophic metastasis rate to other organs was lower for ovarian NEC than for ovarian non-NECs. However, this difference was only statistically significant between endometrial NEC and SC.

### 3.2. Institutional Database

From the institutional database, we identified and retrieved 53 patients with cervical NECs, 16 patients with ovarian NECs, and 5 patients with endometrial NECs. Similar to cases retrieved from the SEER database, uterine cervical NEC was the most common NEC, followed by ovarian NEC and endometrial NEC. Because the numbers of patients with ovarian NEC and endometrial NEC were too small for statistical analysis, we had to exclude them from further evaluation.

Among the uterine cervical carcinomas, we identified and retrieved data on 53 NECs and 3,876 non-NECs (3,206 SCCs and 670 ADCs) (Table 1 and Table S4). Patients with cervical SCC (median 51 years, interquartile range [IQR] 41–61) were significantly (*p* = 0.001 and *p* < 0.001, respectively) older at diagnosis than patients with NEC (median 45 years, IQR 36-53) and patients with ADC (median 46 years, IQR 40–54). There was no significant difference in the median age at diagnosis between patients with cervical NEC and patients with cervical ADC (*p* = 0.145). The overall metastatic rate (percentage of patients with metastasis/total number of patients) was significantly higher in patients with cervical NEC (45.3%, 24/53), followed by patients with cervical ADC (22.8%, 153/670) and patients with cervical SCC (17.5%, 560/3206) (both *p* < 0.001).

**Figure 2:**
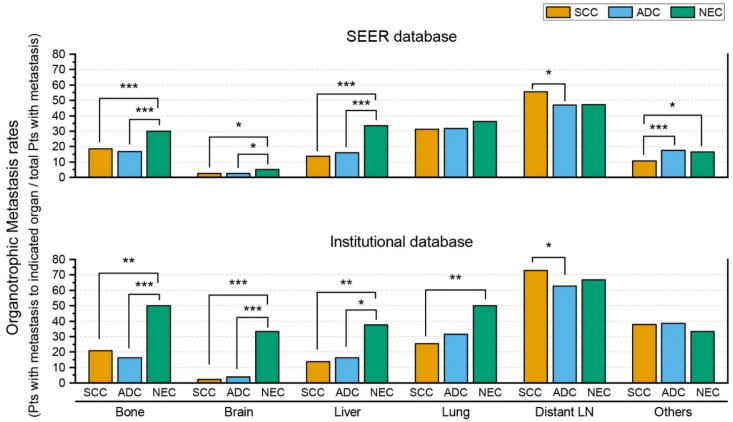
Comparison of organotrophic metastasis patterns of uterine cervical neuroendocrine carcinoma, squamous cell carcinoma, and adenocarcinoma between cases retrieved from the Surveillance, Epidemiology, and End Results (SEER) database and an institutional database. Only statistically significant comparisons are marked with asterisks (*, *p* ≤ 0.05; **, *p* ≤ 0.01; ***, *p* ≤ 0.001). (Abbreviations: SCC, squamous cell carcinoma; ADC, adenocarcinoma; NEC, neuroendocrine carcinoma; Pts, patients; LN, lymph node).

We calculated and compared the organotrophic metastasis rates (the percentages of patients with metastasis to the indicated organ/the total number of patients with metastasis) as we did previously. The results are illustrated in Figure 2. Similar to the cases retrieved from the SEER database, cervical NEC showed significantly higher bone, brain, and liver organotrophic metastasis rates than cervical SCC and cervical ADC. Cervical SCC showed a significantly higher distant LN organotrophic metastasis rate than ADC. However, there were also some differences between the results obtained from the institutional database and those from the SEER database. The organotrophic metastasis rates to other organs were not significantly different between cervical NEC and non-NEC, including SCC and ADC. Cervical NEC showed a significantly higher lung organotrophic metastasis rate than cervical non-NECs. Additionally, cervical NEC showed significantly higher organotrophic metastasis rates to the pancreas and soft tissue than cervical non-NECs.

When we compared the results obtained from the institutional database with those from the SEER database, the overall metastatic rate of cervical NECs from the SEER database (43.2%) was not significantly different from that of cervical NECs from the institutional database (45.3%) (*p* = 0.775). NEC cases from the institutional database showed significantly (*p* = 0.047, *p* < 0.001, and *p* = 0.040, respectively) higher organotrophic metastasis rates to the bone, brain, and other organs than NEC cases from the SEER database. There were no significant differences in liver, lung, and distant LN organotrophic metastasis rates between NEC cases from the institutional database and SEER database. In contrast, non-NECs from the institutional database, including SCC and ADC, showed significantly (both *p* < 0.001) higher overall metastatic rates than SCC and ADC from the SEER database. Non-NEC (SCC and ADC) cases showed no significant differences in bone, brain, and liver organotrophic metastasis rates between the institutional database and SEER database. SCC from the institutional database showed a significantly (*p* = 0.775) lower lung organotrophic metastasis rate than SCC from the SEER database. Both SCC and ADC from the institutional database showed significantly higher organotrophic metastasis rates to distant LNs and other organs than SCC and ADC from the SEER database.

## 4. Discussion

Cervical NEC was the most common NEC in this study. All NECs included in this study showed significantly higher overall metastatic rates than non-NECs of the same primary sites. These findings are consistent with previous reports on NECs in the female genital tract [2,7,8].

All uterine cervical, endometrial, and ovarian NECs showed a higher tendency for bone, brain, and liver organotrophic metastasis than non-NECs of the same primary sites. To our knowledge, no previous study has reported these findings. However, similar findings can be found in some previous studies regarding pulmonary and gastrointestinal NENs. Several previous studies have reported that all pulmonary and gastroenteropancreatic NENs show a higher tendency for liver organotrophic metastasis [3,4,9]. Considering that a higher tendency for liver organotrophic metastasis was also identified in gynecologic NECs in this study, a higher tendency for liver organotrophic metastasis might be a common feature of all NENs in the human body. A previous study reported that rectal neuroendocrine tumors showed a higher tendency for bone organotrophic metastasis [9]. Considering that the rectum, uterus, and ovaries are all located in the pelvic cavity, this finding is quite interesting. Unfortunately, we were unable to find a previous study reporting NENs showing a higher tendency for brain organotrophic metastasis, and this might be a unique feature of NECs in the female genital tract.

We analyzed an institutional database to validate the results obtained from the SEER database. The results obtained from the institutional database confirmed the higher tendency for bone, brain, and liver organotrophic metastasis demonstrated by the uterine cervical NEC cases retrieved from the SEER database. Unfortunately, we were unable to validate the results obtained for endometrial and ovarian NEC cases retrieved from the SEER database because the numbers of patients with endometrial NEC and ovarian NEC retrieved from the institutional database were too small for statistical analysis. A multi-institutional study might be needed to validate these results.

The SEER database is composed of patient data collected from the United States population and only includes the records of metastasis detected at the time of diagnosis. In contrast, the institutional database used in this study is composed of patient data collected from the South Korean population and includes all records of metastasis during the follow-up period. In this study, we thought that a comparison of cervical NEC cases retrieved from the SEER database and cases retrieved from the institutional database might reveal some interesting findings. Cervical SCC and ADC cases retrieved from the institutional database showed significantly higher overall metastatic rates than the cases retrieved from the SEER database. In contrast, the overall metastatic rate of cervical NEC showed no significant difference between databases. In our opinion, this phenomenon is caused by the short follow-up periods of patients with cervical NECs due to their aggressive clinical behavior.

Comparisons of the organotrophic metastasis rates also showed several interesting findings. Most notably, cervical NEC cases retrieved from the institutional database showed significantly higher bone and brain organotrophic metastasis rates than cervical NEC cases retrieved from the SEER database. There are several possible reasons for this finding. First, this result may be caused by the different populations of each database. As we described previously, each database is composed of a population living in different countries with different genetics and lifestyles. Second, differences in medical insurance could be a possible reason for differences in rates of testing. Bone and brain metastases are generally considered a rare event in patients with carcinoma in the female genital tract [10]. Therefore, clinicians in the United States might be hesitant to recommend testing for brain or bone metastases, especially for patients with financial difficulties. In contrast, clinicians in South Korea would recommend systemic evaluations to patients who are less concerned about their financial burden because of the national medical insurance program. Third, this difference might be caused by the fact that the SEER database only includes records of metastasis at the time of diagnosis. Because the institutional database includes all events of metastasis during the follow-up period, it would naturally show higher organotrophic metastasis rates. Regardless, it is premature to make conclusions solely based on the data included in this study. Further studies are needed.

In conclusion, we demonstrated that uterine cervical, endometrial, and ovarian NECs show significantly higher metastatic rates than non-NECs of the same organs. We also demonstrated that uterine cervical, endometrial, and ovarian NECs show significantly higher tendencies for bone, brain, and liver organotrophic metastasis. Monitoring for bone, brain, and liver metastasis might be needed to ensure proper management of patients with cervical, endometrial, and ovarian NECs.

## Figures and Tables

**Table 1:** Comparison of the rates of organotrophic metastasis among different histologic subtypes of uterine cervical carcinomas retrieved from the Surveillance, Epidemiology, and End Results (SEER) database and an institutional database. Each percentage indicates the percentage of patients with metastasis to the indicated organ divided by the total number of patients with metastasis.

	Histologic Subtype			*p* Value		
	NEC	SCC	ADC	NEC vs. SCC	NEC vs. ADC	SCC vs. ADC
SEER database						
N	495	19947	6517			
Patients with metastasis to the indicated organ/Patients with metastasis						
Bone	63/210 (30%)	466/2502 (18.6%)	109/647 (16.8%)	**<0.001**	**<0.001**	0.297
Brain	11/210 (5.2%)	66/2496 (2.6%)	16/646 (2.5%)	**0.03**	**0.047**	0.812
Liver	70/209 (33.5%)	346/2512 (13.8%)	104/654 (15.9%)	**<0.001**	**<0.001**	0.165
Lung	76/209 (36.4%)	777/2489 (31.2%)	205/646 (31.7%)	0.124	0.215	0.801
Distant LN	43/91 (47.3%)	548/987 (55.5%)	137/292 (46.9%)	0.129	0.955	**0.01**
Other	35/214 (16.4%)	275/2569 (10.7%)	117/674 (17.4%)	**0.041**	0.937	**<0.001**
Institutional database						
N	53	3206	670			
Patients with metastasis to the indicated organ/Patients with metastasis						
Bone	12/24 (50%)	117/560 (20.9%)	25/153 (16.3%)	**0.001**	**<0.001**	0.213
Brain	8/24 (33.3%)	13/560 (2.3%)	6/153 (3.9%)	**<0.001**	**<0.001**	0.265
Liver	9/24 (37.5%)	76/560 (13.6%)	25/153 (16.3%)	**0.004**	**0.024**	0.384
Lung	12/24 (50.0%)	143/560 (25.5%)	48/153 (31.4%)	**0.008**	0.073	0.149
distant LN	16/24 (66.7%)	408/560 (72.9%)	96/153 (62.7%)	0.505	0.711	**0.015**
Other	8/24 (33.3%)	212/560 (37.9%)	59/153 (38.6%)	0.654	0.623	0.874

Bolded text indicates statistically significant at 0.05 level. SEER, Surveillance, Epidemiology, and End Results; SCC, squamous cell carcinoma; ADC, adenocarcinoma; NEC, neuroendocrine carcinoma; LN, lymph node.

**Table 2:** Comparison of the rate of organotrophic metastasis among different histologic subtypes of ovarian carcinomas and endometrial carcinomas retrieved from the Surveillance, Epidemiology, and End Results (SEER) database. Each percentage indicates the percentage of patients with metastasis to the indicated organ divided by the total number of patients with metastasis.

	Histologic Subtype				*p* Value		
	NEC	EC	MC	SC	NEC vs. EC	NEC vs. MC	NEC vs. SC
Ovarian carcinoma							
N	242	4958	2700	24129			
Patients with metastasis to the indicated organ/Patients with metastasis							
Bone	13/84 (15.5%)	13/247 (5.3%)	26/260 (10.0%)	157/7293 (2.2%)	**0.003**	0.169	**<** **0.001**
Brain	5/82 (6.1%)	3/248 (1.2%)	2/259 (0.8%)	32/7283 (0.4%)	**0.025**	**0.01**	**<** **0.001**
Liver	40/83 (48.2%)	68/248 (27.4%)	78/264 (29.5%)	1581/7297 (21.7%)	**<** **0.001**	**0.002**	**<** **0.001**
Lung	19/82 (23.2%)	50/247 (20.2%)	56/258 (21.7%)	1263/7271 (17.4%)	0.573	0.78	0.169
Distant LN	8/33 (24.2%)	12/84 (14.3%)	11/97 (11.3%)	735/3166 (23.2%)	0.198	0.088	0.889
Other	16/33 (48.5%)	50/84 (59.5%)	61/100 (61.0%)	2206/3216 (68.6%)	0.279	0.207	**0.013**
Endometrial carcinoma							
N	173	89637	702	10187			
Patients with metastasis to the indicated organ/Patients with metastasis							
Bone	14/72 (19.4%)	314/2661 (11.8%)	5/33 (15.2%)	125/2243 (5.6%)	**0.049**	0.596	**<** **0.001**
Brain	6/72 (8.3%)	102/2659 (3.8%)	1/33 (3.0%)	15/2238 (0.7%)	0.063	0.429	**<** **0.001**
Liver	18/72 (25.0%)	349/2667 (13.1%)	3/33 (9.1%)	245/2242 (10.9%)	**0.003**	0.058	**<** **0.001**
Lung	17/71 (23.9%)	862/2658 (32.4%)	13/33 (39.4%)	367/2233 (16.4%)	0.131	0.106	0.095
Distant LN	15/32 (46.9%)	303/1300 (23.3%)	1/11 (9.1%)	295/1215 (24.3%)	**0.002**	**0.033**	**0.004**
Other	14/32 (43.8%)	702/1309 (53.6%)	8/12 (66.7%)	899/1218 (73.8%)	0.268	0.176	**<0.001**

Bolded text indicates statistically significant at 0.05 level. NEC, neuroendocrine carcinoma; EC, endometrioid carcinoma; MC, mucinous carcinoma; SC, serous carcinoma; LN, lymph node.

## Data Availability

The data presented in this study are available on request from the corresponding author.

## Supplementary Materials

The following supporting information can be downloaded at: https://www.mdpi.com/article/10.3390/curroncol29100587/s1, Table S1: Metastatic patterns of uterine cervical carcinomas retrieved from the Surveillance, Epidemiology, and End Results (SEER) database; Table S2: Metastatic patterns of ovarian carcinomas retrieved from the Surveillance, Epidemiology, and End Results (SEER) database; Table S3: Metastatic patterns of endometrial carcinomas retrieved from the Surveillance, Epidemiology, and End Results (SEER) database; Table S4: Metastatic patterns of uterine cervical carcinomas retrieved from the institutional database.
