# Supplementary material for: Neuroendocrine Carcinomas of the Uterine Cervix, Endometrium, and Ovary Show Higher Tendencies for Bone, Brain, and Liver Organotrophic Metastases
Source: Curr Oncol. 2022 Oct 6;29(10):7461–9. doi: 10.3390/curroncol29100587 (PMC9600665; doi:10.3390/curroncol29100587)
Supplement: Supplementary file 1 [file curroncol-29-00587-s001.zip › Table S1.pdf]

Supplementary Table S1. Metastatic patterns of uterine cervical carcinomas retrieved from the Surveillance, Epidemiology, and End Results (SEER) database.

	Histologic subtype			*P* value		
	NEC	SCC	ADC	NEC vs SCC	NEC vs ADC	SCC vs ADC
N	495	19947	6517			
Patients with metastasis/Total patients						
	214/495 (43.2%)	2569/19947 (12.9%)	674/6517 (10.3%)	**< 0.001**	**< 0.001**	**< 0.001**
Patients with indicated organ metastasis/Total Patients						
Bone	63/484 (13.0%)	467/19489 (2.4 %)	109/6324 (1.7%)	**< 0.001**	**< 0.001**	**0.002**
Brain	11/484 (2.3%)	66/19483 (0.3%)	16/6321 (0.3%)	**< 0.001**	**< 0.001**	0.293
Liver	70/483 (14.5%)	346/19500 (1.8%)	104/6328 (1.6%)	**< 0.001**	**< 0.001**	0.489
Lung	76/483 (15.7%)	778/19471 (4.0%)	205/6319 (3.2%)	**< 0.001**	**< 0.001**	**0.007**
distant LN	43/200 (21.5%)	551/8015 (6.9%)	137/2802 (4.9%)	**< 0.001**	**< 0.001**	**< 0.001**
Other	35/495 (7.1%)	276/19947 (1.4%)	118/6517 (1.8%)	**< 0.001**	**< 0.001**	**< 0.001**
Patients with indicated organ metastasis/Patients with metastasis						
Bone	63/210 (30%)	466/2502 (18.6%)	109/647 (16.8%)	**< 0.001**	**< 0.001**	0.297
Brain	11/210 (5.2%)	66/2496 (2.6%)	16/646 (2.5%)	**0.03**	**0.047**	0.812
Liver	70/209 (33.5%)	346/2512 (13.8%)	104/654 (15.9%)	**< 0.001**	**< 0.001**	0.165
Lung	76/209 (36.4%)	777/2489 (31.2%)	205/646 (31.7%)	0.124	0.215	0.801
distant LN	43/91 (47.3%)	548/987 (55.5%)	137/292 (46.9%)	0.129	0.955	**0.01**
Other	35/214 (16.4%)	275/2569 (10.7%)	117/674 (17.4%)	**0.041**	0.937	**< 0.001**

Bolded text indicates statistically significant at 0.05 level.
SCC, squamous cell carcinoma; ADC, adenocarcinoma; NEC, neuroendocrine carcinoma
