# Supplementary material for: Neuroendocrine Carcinomas of the Uterine Cervix, Endometrium, and Ovary Show Higher Tendencies for Bone, Brain, and Liver Organotrophic Metastases
Source: Curr Oncol. 2022 Oct 6;29(10):7461–9. doi: 10.3390/curroncol29100587 (PMC9600665; doi:10.3390/curroncol29100587)
Supplement: Supplementary file 1 [file curroncol-29-00587-s001.zip › Table S2.pdf]

Supplementary Table S2. Metastatic patterns of ovarian carcinomas retrieved from the Surveillance, Epidemiology, and End Results (SEER) database.

	Histologic subtype				*P* value					
	NEC	EC	MC	SC	NEC vs EC	NEC vs MC	NEC vs SC	EC vs MC	EC vs SC	MC vs SC
N	242	4958	2700	24129						
Patients with metastasis/Total patients										
	87/217 (40.1%)	259/4946 (5.2%)	274/2687 (10.2%)	7563/24028 (31.5%)	**< 0.001**	**< 0.001**	**0.007**	**< 0.001**	**< 0.001**	**< 0.001**
Patients with indicated organ metastasis/Total Patients										
Bone	14/233 (6.0%)	13/4904 (0.3%)	26/2648 (1.0%)	158/23525 (0.7%)	**< 0.001**	**< 0.001**	**< 0.001**	**< 0.001**	**0.001**	0.07
Brain	6/230 (2.6%)	3/4905 (0.1%)	2/2647 (0.1%)	32/23515 (0.1%)	**< 0.001**	**< 0.001**	**< 0.001**	1.000	0.174	0.575
Liver	44/232 (19.0%)	68/4906 (1.4%)	78/2650 (2.9%)	1588/23528 (6.7%)	**< 0.001**	**< 0.001**	**< 0.001**	**< 0.001**	**< 0.001**	**< 0.001**
Lung	24/230 (10.4%)	50/4906 (1.0%)	57/2644 (2.2%)	1267/23494 (5.4%)	**< 0.001**	**< 0.001**	**0.001**	**< 0.001**	**< 0.001**	**< 0.001**
distant LN	12/97 (12.4%)	12/1952 (0.6%)	11/1067 (1.0%)	746/9267 (8.0%)	**< 0.001**	**< 0.001**	0.116	0.209	**< 0.001**	**< 0.001**
Other	19/97 (19.6%)	50/1952 (2.6%)	63/1070 (5.9%)	2248/9308 (24.2%)	**< 0.001**	**< 0.001**	0.296	**< 0.001**	**< 0.001**	**< 0.001**
Patients with indicated organ metastasis/Patients with metastasis										
Bone	13/84 (15.5%)	13/247 (5.3%)	26/260 (10.0%)	157/7293 (2.2%)	**0.003**	0.169	**< 0.001**	**0.045**	**0.001**	**< 0.001**
Brain	5/82 (6.1%)	3/248 (1.2%)	2/259 (0.8%)	32/7283 (0.4%)	**0.025**	**0.01**	**< 0.001**	0.618	0.107	0.327
Liver	40/83 (48.2%)	68/248 (27.4%)	78/264 (29.5%)	1581/7297 (21.7%)	**< 0.001**	**0.002**	**< 0.001**	0.594	**0.031**	**0.002**
Lung	19/82 (23.2%)	50/247 (20.2%)	56/258 (21.7%)	1263/7271 (17.4%)	0.573	0.78	0.169	0.687	0.242	0.072
distant LN	8/33 (24.2%)	12/84 (14.3%)	11/97 (11.3%)	735/3166 (23.2%)	0.198	0.088	0.889	0.553	0.055	**0.006**
Other	16/33 (48.5%)	50/84 (59.5%)	61/100 (61.0%)	2206/3216 (68.6%)	0.279	0.207	**0.013**	0.838	0.078	0.108

Bolded text indicates statistically significant at 0.05 level.
NEC, neuroendocrine carcinoma; EC, endometrioid carcinoma; MC, mucinous carcinoma; SC, serous carcinoma; LN, lymph node
