# Supplementary material for: Neuroendocrine Carcinomas of the Uterine Cervix, Endometrium, and Ovary Show Higher Tendencies for Bone, Brain, and Liver Organotrophic Metastases
Source: Curr Oncol. 2022 Oct 6;29(10):7461–9. doi: 10.3390/curroncol29100587 (PMC9600665; doi:10.3390/curroncol29100587)
Supplement: Supplementary file 1 [file curroncol-29-00587-s001.zip › Table S3.pdf]

Supplementary Table S3. Metastatic patterns of endometrial carcinomas retrieved from the Surveillance, Epidemiology, and End Results (SEER) database.

	Histologic subtype				*P* value					
	NEC	EC	MC	SC	NEC vs EC	NEC vs MC	NEC vs SC	EC vs MC	EC vs SC	MC vs SC
N	173	89637	702	10187						
Patients with metastasis/Total patients										
	73/165 (44.2%)	2725/89334 (3.1%)	33/699 (4.7%)	2291/10152 (22.6%)	**< 0.001**	**< 0.001**	**< 0.001**	**0.011**	**< 0.001**	**< 0.001**
Patients with indicated organ metastasis/Total Patients										
Bone	16/170 (9.4%)	314/88478 (0.4%)	5/685 (0.7%)	125/9992 (1.3%)	**< 0.001**	**< 0.001**	**< 0.001**	0.101	**< 0.001**	0.229
Brain	6/170 (3.5%)	103/88478 (0.1%)	1/686 (0.1%)	15/9990 (0.2%)	**< 0.001**	**< 0.001**	**< 0.001**	0.552	0.356	1.000
Liver	20/170 (11.8%)	351/88490 (0.4%)	3/686 (0.4%)	246/9995 (2.5%)	**< 0.001**	**< 0.001**	**< 0.001**	0.756	**< 0.001**	**0.001**
Lung	18/169 (10.7%)	865/88480 (1.0%)	13/687 (1.9%)	369/9983 (3.7%)	**< 0.001**	**< 0.001**	**< 0.001**	**0.016**	**< 0.001**	**0.014**
distant LN	18/74 (24.3%)	306/38929 (0.8%)	1/257 (0.4%)	296/5060 (5.8%)	**< 0.001**	**< 0.001**	**< 0.001**	0.727	**< 0.001**	**< 0.001**
Other	15/74 (20.3%)	703/38933 (1.8%)	8/258 (3.1%)	901/5064 (17.8%)	**< 0.001**	**< 0.001**	0.580	0.15	**< 0.001**	**< 0.001**
Patients with indicated organ metastasis/Patients with metastasis										
Bone	14/72 (19.4%)	314/2661 (11.8%)	5/33 (15.2%)	125/2243 (5.6%)	**0.049**	0.596	**< 0.001**	0.584	**< 0.001**	**0.037**
Brain	6/72 (8.3%)	102/2659 (3.8%)	1/33 (3.0%)	15/2238 (0.7%)	0.063	0.429	**< 0.001**	1	**< 0.001**	0.209
Liver	18/72 (25.0%)	349/2667 (13.1%)	3/33 (9.1%)	245/2242 (10.9%)	**0.003**	0.058	**< 0.001**	0.793	**0.021**	1
Lung	17/71 (23.9%)	862/2658 (32.4%)	13/33 (39.4%)	367/2233 (16.4%)	0.131	0.106	0.095	0.396	**< 0.001**	**0.002**
distant LN	15/32 (46.9%)	303/1300 (23.3%)	1/11 (9.1%)	295/1215 (24.3%)	**0.002**	**0.033**	**0.004**	0.474	0.567	0.477
Other	14/32 (43.8%)	702/1309 (53.6%)	8/12 (66.7%)	899/1218 (73.8%)	0.268	0.176	**< 0.001**	0.367	**< 0.001**	0.525

Bolded text indicates statistically significant at 0.05 level.
NEC, neuroendocrine carcinoma; EC, endometrioid carcinoma; MC, mucinous carcinoma; SC, serous carcinoma; LN, lymph node
