# Supplementary material for: Neuroendocrine Carcinomas of the Uterine Cervix, Endometrium, and Ovary Show Higher Tendencies for Bone, Brain, and Liver Organotrophic Metastases
Source: Curr Oncol. 2022 Oct 6;29(10):7461–9. doi: 10.3390/curroncol29100587 (PMC9600665; doi:10.3390/curroncol29100587)
Supplement: Supplementary file 1 [file curroncol-29-00587-s001.zip › Table S4.pdf]

Supplementary Table S4. Metastatic patterns of uterine cervical carcinomas retrieved from the institutional database.

	Histologic subtype			*P* value		
	NEC	SCC	ADC	NEC vs SCC	NEC vs ADC	SCC vs ADC
Total patients' number	53	3206	670			
Age at diagnosis	45 (36-53)	51 (41-61)	46 (40-54)	**0.001**	0.145	**< 0.001**
Patients with metastasis/Total patients						
	24/53 (45.3%)	560/3206 (17.5%)	153/670 (22.8%)	**< 0.001**	**< 0.001**	**0.001**
Patients with indicated organ metastasis/Total Patients						
Bone	12/53 (22.6%)	117/3206 (3.6%)	25/670 (3.7%)	**< 0.001**	**< 0.001**	0.918
Brain	8/53 (15.1%)	13/3206 (0.4%)	6/670 (0.9%)	**< 0.001**	**< 0.001**	0.121
Liver	9/53 (17.0%)	76/3206 (2.4%)	25/670 (3.7%)	**< 0.001**	**< 0.001**	**0.044**
Lung	12/53 (22.6%)	143/3206 (4.5%)	48/670 (7.2%)	**0.001**	**< 0.001**	**0.003**
distant LN	16/53 (30.2%)	408/3206 (12.7%)	96/670 (14.3%)	**0.002**	**< 0.001**	0.262
Other	8/53 (15.1%)	212/3206 (6.6%)	59/670 (8.8%)	0.138	**0.024**	**0.043**
Patients with indicated organ metastasis/Patients with metastasis						
Bone	12/24 (50%)	117/560 (20.9%)	25/153 (16.3%)	**0.001**	**< 0.001**	0.213
Brain	8/24 (33.3%)	13/560 (2.3%)	6/153 (3.9%)	**< 0.001**	**< 0.001**	0.265
Liver	9/24 (37.5%)	76/560 (13.6%)	25/153 (16.3%)	**0.004**	**0.024**	0.384
Lung	12/24 (50.0%)	143/560 (25.5%)	48/153 (31.4%)	**0.008**	0.073	0.149
distant LN	16/24 (66.7%)	408/560 (72.9%)	96/153 (62.7%)	0.505	0.711	**0.015**
Other	8/24 (33.3%)	212/560 (37.9%)	59/153 (38.6%)	0.654	0.623	0.874
Pancreas	3/24 (12.5%)	4/560 (0.7%)	1/153 (0.7%)	**0.002**	**< 0.001**	1
Soft tissue	4/24 (16.7%)	15/560 (2.7%)	5/153 (3.3%)	**0.006**	**0.021**	0.782

Bolded text indicates statistically significant at 0.05 level.
SCC, squamous cell carcinoma; ADC, adenocarcinoma; NEC, neuroendocrine carcinoma
